# Bioethics and medical/legal considerations on cochlear implants in children

**DOI:** 10.1590/S1808-86942012000300013

**Published:** 2015-10-14

**Authors:** Ivan Dieb Miziara, Carmen Silvia Molleis Galego Miziara, Robson Koji Tsuji, Ricardo Ferreira Bento

**Affiliations:** a Professor of Forensic Medicine and Deontology in the Medical School of ABC; Assisting Professor of Forensic Medicine and Bioethics in the Medical Sciences School at the Santa Casa de São Paulo (Professor of Forensic Medicine and Deontology in the Medical School of ABC).; b Pediatric Neurologist, PhD in Sciences in the Medical School at USP (Pediatric Neurologist at the Hospital das Clínicas da FMUSP).; c PhD in Sciences in the Medical School at USP, ENT at the Hospital das Clínicas da FMUSP (MD, member of the cochlear implant in the ENT Clinic at HCFMUSP).; d Professor of Otorhinolaryngology in the Medical School at USP (Director of the ENT Clinical Division at HCFMUSP). Hospital das Clínicas da Faculdade de Medicina da USP.

**Keywords:** bioethics, cochlear implants, forensic medicine

## Abstract

Cochlear implants are the best treatment for congenital profound deafness. Pediatric candidates to implantation are seen as vulnerable citizens, and the decision of implanting cochlear devices is ultimately in the hands of their parents/guardians. The Brazilian Penal Code dictates that deaf people may enjoy diminished criminal capacity. Many are the bioethical controversies around cochlear implants, as representatives from the deaf community have seen in them a means of decimating their culture and intrinsic values.

**Objective:**

This paper aims to discuss, in bioethical terms, the validity of implanting cochlear hearing aids in children by analyzing their vulnerability and the social/cultural implications of the procedure itself, aside from looking into the medical/legal aspects connected to their criminal capacity.

**Materials and Methods:**

The topic was searched on databases Medline and Lilacs; ethical analysis was done based on principialist bioethics.

**Results:**

Cochlear implants are the best therapeutic option for people with profound deafness and are morally justified. The level of criminal capacity attributed to deaf people requires careful analysis of the subject's degree of understanding and determination when carrying out the acts for which he/she has been charged.

**Conclusion:**

Cochlear implants are morally valid. Implantations must be analyzed on an each case basis. ENT physicians bear the ethical responsibility for indicating cochlear implants and must properly inform the child's parents/guardians and get their written consent before performing the procedure.

## INTRODUCTION

The vulnerability of patients involved in research or submitted to new therapeutic/ diagnostic procedures is an important matter, particularly for ENTs. For the purposes of this paper, vulnerability is to be understood as the set of cultural, social, ethnic, political, economic, educational, or health status differences established within a certain group in relation to society that result in the discrimination of such group by others. Vulnerability impairs the ability of those affected by it to freely express their will. Children with profound deafness picked as candidates for cochlear implants undoubtedly account for a vulnerable group.

This group of patients may be categorized as vulnerable not only because they are children - thus imbued with diminished capacity and decision-making power - but because of the handicap that will accompany them into adult age. It should be noted that in the Brazilian legal system the deaf may be considered partially criminally capable or even criminally incapable. Given the presence of hearing loss and reduced cognitive skills, the legal system sees them as criminally incapable (or subject to lesser punishment) as they assumedly cannot understand the illegality of the acts they may perpetrate.

Exacerbated vulnerability leads to a reduction or complete loss of individual freedom and autonomy, as the same factors that lead one into a vulnerable condition prevent him/her from exerting his/her free will. In such cases, it is the patients' guardians who make the call on implanting cochlear hearing aids or not. However, it is worthwhile pointing out that the physician, from the standpoint of ethics, must be able to offer the patient the various possibilities pertaining to his/her case. Current medical literature states that the better treatment for children with deafness or profound sensorineural hearing loss seems to be the early use of cochlear implants^1^. Nonetheless, other options such as ordinary hearing aids (when applicable) and Brazilian sign language (LIBRAS) must be mentioned to the patient's guardians, as pediatric patients are not allowed to decide for themselves. The patients' vulnerability, boosted by his/her handicap, may be exacerbated by unfavorable social/economic circumstances.

Conflict is often present in such choices. On one side, there are those who analyze the issue of deafness purely from a medical neurophysiological perspective, for whom hearing loss is the outcome of auditory disease with specific histologic and cytologic disorders. On the other side, there is the community of deaf people who rely on a different form of communication - mostly sign language - to express themselves. This is a community with a specific set of costumes, values, and attitudes. For the members of this community, deafness per se is not associated with low scores on nonverbal intelligence tests (or any other cognition test). Therefore, for them, hearing loss is not a handicap. Activists in the deaf community (especially in the United States) have established clear definitions around what they call the “social construct of deafness,” and see the handicap as a variation of normality^2^.

This paper aims to offer a literature review on the matter, and discuss the issues seen in Brazil from the standpoint of bioethics around the decision related to opting for or against the placement of cochlear implants in pediatric profound deafness patients. The role of ENTs, and the social-cultural implications related to the decision on cochlear implants is also discussed herein. Additionally, as a secondary goal, this paper proposes an analysis on the medical and legal aspects connected to the criminal capacity of deaf individuals.

## METHOD

A search for papers was done on databases Medline and Lilacs, using keywords “ethics”, “bioethics”, and “cochlear implant” and covering the period from 2005 to 2001. The discussion of bioethics was based on Beauchamp and Childress' biomedical ethics.

## RESULTS

### Impact on the opinion of the patients' guardians

Congenital hearing loss affects approximately 1 out of every 1,000 live births, and is inherited in 50% of the cases. At least 20 genes have been found to bear relations with non-syndromic recessive hearing loss, i.e., deafness not associated with other clinical findings. Hearing loss is a long-term public health concern. Genetic cases may lead to progressive hearing loss starting in early childhood^3^. From the medical standpoint, deafness in children and adults is a disease deserving proper treatment^4^.

Hardonk et al.^3^ have divided patient guardians into three groups: 1) those who highly appreciate the development of the patient's oral communication skills and compare it to the alternatives of cochlear implant and conventional hearing aids (and the LIBRAS sign language in the case of Brazil); 2) those for whom the risks inherent to surgery are more relevant, thus disregarding medical advice as to the superiority of cochlear implants; and 3) those who simply accept the advice on cochlear implants without questioning it. The authors concluded that ENTs must be aware of the impact hey may have in the decision made by the patients' guardians, and carefully analyze each case based on ethical standards before offering the various therapeutic options available.

According to Kermit^5^, two mutually excluding approaches must be considered in the postoperative care of implant cochlear patients: the bilingual approach - sign and spoken language; and the monolingual approach with spoken language alone. The author further states that despite the scientific uncertainties surrounding both doctrines, it is possible that some damage be produced in children rehabilitated using the monolingual approach. It is thus recommended that the “principle of precaution” be adopted and that patients be offered the bilingual approach.

### Allocation of public funds

Another problem of bioethical relevance pertaining to cochlear implants relates to the allocation of public funds^6^ for research and/ or treatment purposes - a pressing issue in developing countries such as Brazil. In these cases, it seems the best option would be to set up cochlear implant programs focused on the needs and benefits for the patients. Systems based on waiting lines (in first come first serve basis) or on the assignment of priority to socially/economically less favored patients do not seem not to be the best option^6^.

Unfortunately, in Brazil this problem is far from being completely solved. Cochlear implants are paid in full by the public health care system (SUS), but waiting lines still exist. Conventional hearing aids are also covered, but waiting lines are exceedingly long.

The benefits yielded on individual basis with the use of cochlear implants (enhanced ease on spoken language acquisition, better integration to the hearing world, and better overall quality-of-life) are also reflected upon society as a whole. The life long cost of treating a child with congenital hearing loss in the United States has been estimated at one million US dollars, and includes special education needs, support/ social services, and reduced productivity levels seen in deaf adults^1^.

### Cultural aspects

The cultural aspects at play must also be considered, the most important being the issues related to the deaf community. According to Levy^7^, this discussion presents interests that expand beyond specific points of conflict, as it derives from a topic filled with controversy and contradiction: the value of culture. To Levy, some of our institutions support the idea that cultures carry intrinsic value, without analyzing whether these values bring benefits to the members of such culture. On the other hand, at times we believe that cultures carry only sentimental value or, in other words, that value is important only as it helps the members of the community satisfy their specific needs and live their lives unbothered. The implication of intuitive thought is that the patient's individual liberty and autonomy cannot be limited on behalf of the integrity of a certain culture. According to Levy, as long as our intuitive thought on the value of a certain culture are confusing and contradictory, we will not be able to deal with the conflicts that set apart individual preferences and the needs of a given culture^7^.

### The deaf culture

In the specific case of cochlear implants, individual needs and preferences are set against those of the deaf culture. Discussions here revolve around the clearly separated instrumental aspects and intrinsic concepts surrounding the values of the deaf culture^7^.

It is possible to maintain what Levy referred to as the deaf culture if the benefits of new technologies - such as cochlear implants - do not reach the deaf community. Levy says we are faced with a predicament: whether we are benefitting children by giving them the means to belong to the hearing community at the expense not of some individuals, but of the deaf culture to which these children belonged. He concludes by saying that if cultures have only instrumental value - assuming that the deaf culture is a genuine culture - then there is no ethical problem in allowing them to perish. Levy adds that if cultures have intrinsic value, the deaf culture should not be allowed to perish^7^.

Why, and based on what, would the deaf culture oppose cochlear implants by any degree?

Three basic points are used to that end. First, the deaf community refuses to accept that hearing loss is a disease. Consequently, it should not be treated by means of medical or surgical intervention. Levy named it the disability argument^7^. Second, treating hearing loss through medical intervention is offensive for the deaf, as it implies they are inferior to the non-deaf. This is the message argument. Lastly, whether it is a handicap, deafness is the means to access a vivid, rich culture. As all cultures have intrinsic value, actions leading to their demise should not be pursued. This is the cultural argument.

In his study, Levy carefully examines each one of these arguments. Regarding the disability argument, the author finds it is consistent with the statement that the deaf culture has intrinsic value; it is not a disability, once it grants access to a whole culture. We believe this is not a valid argument, given that deafness is not analyzed in an isolated fashion considering its neurophysiological characteristics as studied by physicians, but rather within a context in which patients are blessed with some cultural value that magically sweeps away all neuron injury. Acoustic nerve injury is not fiction. It is instead a reality that crushes the arguments of deaf culture activists, who argue that despite the handicap deafness is not a disability, as the handicaps associated with it would not be natural, but rather social in their origin^7^. Being that the case, say the activists, we should treat society instead of the deaf.

We cannot accept these arguments nor their intention of granting the deaf a status similar to that of belonging to an ethnic group such as people of African, native, Arab, or Jewish descent. It is obvious that the deaf face numerous handicaps (discrimination, low level of education, reduced life expectancy, higher unemployment rates etc) and enjoy a few advantages, if they may be called so, such as partial criminal capacity. These handicaps and advantages are not natural, and nor is the inevitable consequence of their individual traits or ethnicity, although we acknowledge they are social in their origin. As Levy says, blacks bear similar handicaps not because they are blacks, but because they live in a society that discriminates against them^7^. The consequences of such rationale are within the reach of the simplest logic: one cannot eliminate the disadvantages experienced by blacks by eliminating their blackness.

Another consequence of this rationale is that disadvantages may be categorized strictly by social reasons, as long as such handicaps meet two basic criteria:

1. changes in social arrangements lead to the immediate elimination of the handicap;
2. there is no reason why changes in social arrangements cannot occur.

### Social arrangements

Levy presents interesting examples of handicaps introduced (or worsened) by social arrangements meeting these criteria. One might say that people on wheelchairs are socially disabled as buildings are equipped with stairs instead of ramps. This argument satisfies both conditions: the social arrangement may be changed so that public buildings are no longer equipped with stairs and there is no reason why a regulation forcing engineers and architects to design and build facilities equipped with ramps for wheelchair users instead of stairs is not passed.

We must acknowledge that part of the handicaps faced by the deaf are of a social nature. Oliver Sacks^8^ described brilliantly the nefarious consequences of the ban placed on sign language in the Milan Congress in 1880. The deaf were deprived not only of their language, but of any language at all.

Despite protests from activists in the deaf community, Levy also describes natural handicaps the deaf experience: they cannot hear car horns while crossing streets; they cannot hear fire alarms, etc. Fire alarm systems cannot be changed to use light bulbs, for instance, given that hearing - differently from seeing - has a greater impact including when we are asleep. Therefore, it seems there are no reasonable compelling justifications to refrain physicians from repairing this natural disability with the available medical resources.

## DISCUSSION

### Bioethical analysis

How can we analyze the choice made by the guardians of a deaf child for the placement of a cochlear implant in bioethical terms? Various methods can be employed - from case series analysis to considerations on the principles described by Beauchamp and Childress (Autonomy, Beneficence, Non-maleficence, and Justice)^9^.

If the latter mode of analysis is chosen, a problem is immediately posed: pediatric patients are vulnerable, their autonomy is diminished by their condition, and the responsibility over the final decision on placing cochlear implants belongs to their guardians. ENTs must be careful enough not to adopt a paternalist stance in such situations.

Cochlear implants are the best option available today to rehabilitate profoundly deaf children meeting the criteria for implantation^1^. Therefore the principles of beneficence and non-maleficence are satisfied by cochlear implants. However, as the final decision lies in the hands of the patients' guardians, both principles are subject to the personal beliefs of the decision makers concerning the arguments presented by Levy. Factors such as surgery risk, waiting time until surgery (along with the developmental delays and cognitive losses experienced by the child in the meantime), etc. These aspects call for the principle of Justice, principally as the allocation of public funds and the ensuing consequences upon the public health care in Brazil are taken into account.

The money spent on one single cochlear implant could be used to pay for a much higher number of conventional hearing aids. Clearly, a massive implantation program covering children with indication for cochlear implants is far from being given priority in Brazil. Besides, public funds are scarce and waiting lines will be long, and only people who cannot afford the procedure will be in the waiting lines. Alternatives to such program also present similar challenges. For example: if sign language is picked as the solution for the problem, public schools will have to hire LIBRAS interpreters for every classroom where there is a student with hearing loss, leading to significant increases in the expenditures of the Brazilian public education system. As mentioned above, the lifelong public expenditure with each congenitally deaf child in the US amounts to about one million dollars^1^.

Social disparities in the supply of health care services bear relevant ethical implications and may be considered with the aid of the philosophical principles of distributive justice. Norman Daniels, using John Rawls' seminal ideas, argues that a just society offers equal opportunity to all its members. A central point for the author is the thesis on the moral importance of preventing and treating diseases and disabilities. In more concrete terms, health care systems contribute to the supply of equal opportunity by offering treatment and preventive care to everyone^10^, ^11^.

After this brief analysis, we have reached a point of convergence with the critics of biomedical ethics ^12^: which is the most important of the four principles? There appears to be no answer for this question, as we were unable to even properly resolve the issue of autonomy for vulnerable populations. The answer could also lie in the current recommendations for post-implantation rehabilitation accepted by the ENT community, i.e., in the adoption of a bilingual system covering both communication possibilities for the treated children. As this approach is adopted, the issues related to the disappearance of the deaf culture are automatically minimized, while children with profound deafness are provided with the best treatment medical care can offer and given the choice, as they become adults, of picking the community they wish to belong to.

This rationale may not win over the defenders of the deaf culture, and it is possible that the best approach is to use another analytical model, i.e., the case series-based method, to analyze each individual case through paradigms. Case series-based bioethical analysis may be the most adequate form of reflecting on the ethics of the issue at hand, mainly when considering two types of parents/guardians of deaf children: those with normal hearing and those with hearing loss. Parents with normal hearing experience enormous pain when they find their children are deaf. They definitely see deafness as a disability. They may be acquainted with the social manifestations of deaf culture, but not with deaf culture itself, and tend to intuitively seek a medical solution for their case.

There is nothing wrong with seeking a solution, and the pain they experience is perfectly understandable. Albeit in another context, Gilles Deleuze has said that despite the way language is acquired, the elements of language are given together, all at once, as they do not exist independently from their possible differential relations^13^.

Non-deaf parents/guardians of deaf children have an inherent difficulty in participating in the language acquisition efforts of their children, possibly the most relevant factor in parent/child communication. However, as parents/guardians do not accept their children's condition and opt for implantation without further consideration, they may accentuate their children's vulnerability as this option does not take other alternatives for their children into account. Therefore, it is important that parents/ guardians are aware of the origin of their difficulty accepting the deafness of their children, so that a conscious decision considering their children first is made.

Two aspects are at play when deaf parents/guardians consider offering cochlear implants to their children: to them, the fact of having a deaf child is not seen as a pain point, given that they live with the condition and have adapted to it. Perhaps for these parents/guardians the decision of offering cochlear implants to their children is not relevant. The second aspect relates to parents/guardians members of the deaf community who highly appreciate the deaf culture, i.e., parents/guardians who reject implantation a priori, given that as their children are offered cochlear implants they become members of another culture, thus generating in parents/guardians a more tangible sense of loss.

In both cases the vulnerability of deaf children is exacerbated. One might say (assuming cochlear implants benefit deaf children) that the decisions made by the parents/guardians are more based on their own ideas on the matter than on the future benefits their children may enjoy. This situation must be observed with caution by ENTs and by the multidisciplinary team that follows patients in cochlear implant programs. Psychological assessment of the parents/guardians must be extremely accurate to avoid precipitous decisions that cannot be changed in the future.

### Informed consent

Informed consent forms must be signed by the parents/guardians of pediatric patients and by adult patients as well, so that the procedure gains minimum moral validity^14^, ^15^, ^16^. Hyde & Power^15^ have found that informed consent forms used in these cases focus almost exclusively on the medical matters concerning surgery risk. The authors state that such document is aimed solely at protecting the physicians in the event of accidents, negligence, and unsatisfactory outcomes.

Parents/guardians usually have high expectations for the outcome of the procedure in improving areas such as communication, education, socialization, and future employment possibilities for their children^16^. Unmet expectations may lead to conflict. Parents/guardians often say, “What if the implant does not work?” For Hyde & Power^15^ this type of question carries another meaning in it: “What can be done if the implant does not meet our initial expectations?” In other words, “What can you do if the outcome of the implant is not GREAT?”

As a consequence, may cochlear implantation programs include the following message in their informed consent forms: “We cannot predict with certainty or assure the level of speech recognition that the subject will show once equipped with a cochlear implant”.

Other aspects also require consideration. One of them is that “deaf life”^14^ may be fulfilling and satisfactory without cochlear implants, and that the promises of a normal life made by cochlear implant proponents may be regarded as unnecessary. Thus, it seems reasonable to suggest that informed consent forms for parents/ guardians of deaf children should be amended to included explanations on the social, linguistic, and cultural factors inherent to being deaf. In other words, instead of looking only at the disadvantages of being deaf, the viability of deaf life should also be presented to parents/ guardians. This is a path with no return. Once implanted, individuals lose access to the personal, social, cultural, and linguistic wealth offered by the deaf community. Many authors have wondered whether this information is truly conveyed to parents/guardians before implantation^15^, ^16^, ^17^. We believe it is not. There is a natural tendency among physicians of not recognizing the vitality of deaf life^17^, and how much that could mean to affected individuals, for instance, in maturing emotionally (as they overcome obstacles despite difficulties) and growing as a person living in adverse hostile conditions. Physicians usually pass on to parents/guardians the so-called “medical perspective on deafness.”

Convincing non-deaf parents/guardians of the existence of such cultural wealth and vitality is a tall order. Nonetheless, this difficulty should not serve as excuse for physicians not to present the facts to their patients' parents/guardians . Informed decisions can only be made once all facts are presented.

### Medical and legal aspects

The main consequence of deafness relates to the communication difficulties faced by individuals with hearing loss and the consequent adverse impacts felt upon various aspects of their global development. The speech acquisition and language development processes of children with hearing loss are negatively affected, thus impairing their ability of communicating and receiving information through oral language oral. Deafness brings implicit involvements related to the formation of concepts and abstraction, besides affecting the characterization of one's personality, sense of identification, and social integration.

According to Lacan, language and its structure are preexisting factors as subjects enter each mental development stage. In other words, language produces a type of “incision” into one's mental development, elevating him/ her to a state of full capacity. The Brazilian legal system sees the deaf as a special class of individuals. In the Penal area (Article 26 in the Brazilian Penal Code), similarly to the Civil code and according to jurisprudence, people with disabilities may be assigned partial or no criminal capacity, as long as their mental development has been compromised on absolute terms^18^.

Barros^18^ has described three situations concerning the criminal capacity of the deaf:

1. deaf persons with no self-determination capability when the crime was committed: the defendant is seen as a mentally ill person and given the same treatment as someone with oligophrenia (article 26, head provision, Penal Code);
2. deaf persons with diminished self-determination capability when the crime was committed: the defendant is seen as someone with partial criminal capacity and treated as per Paragraph One Article 26;
3. deaf persons with full self-determination capability when the crime was committed: the defendant is seen as criminally capable and subject to a regular trial.

Intermediate cases are granted relative criminal capacity based on the subject's comprehension and self-determination levels. The Law sees hearing loss as an impediment to one's complete mental development (deaf subjects are assumed to have reduced cognitive skills) and thus patients are deemed partially or totally unable to understand the legal norm and comply with it.

The impending question here revolves around how individuals with cochlear implants should be considered. If they are now able to hear, have they ceased to belong to the class of deaf people?

An even more relevant question from the medical/legal standpoint is whether children undergoing early treatment for hearing loss with cochlear implants will be able to realize their “full development” as defined by Law. As discussed above, the deaf community does not see hearing loss as a disability or as a factor associated with poor scores in nonverbal intelligence tests (and other cognitive function tests). Activists in the deaf community have established a clear distinction between the social construction of deafness and see hearing loss as a variant from normality^2^. Clearly the Brazilian Penal Code - written in the 1940s - is not current enough and offers a diverse understanding on the matter at hand.

We believe that deaf individuals will never cease to carry their condition, even if they use conventional hearing aids or cochlear implants. It is yet unknown to what degree individuals using cochlear implants will develop mentally and cognitively. Individuals must be assessed on an each case basis (considering numerous factors such as age of implantation, adopted rehabilitation approach and other factors that certainly have affected their mental development). It is implied that the criminal capacity of deaf subjects on trial for committing a crime must be analyzed separately for each case through adequate hearing and forensic neurophysiological tests to find out to what extent the defendant had a grasp of social norm when the crime was committed, as criminal capacity must be analyzed vis-à-vis the time when the crime was perpetrated.

## CONCLUSION

Cochlear implants pose relevant dilemmas. The first relates to the definition used for disability. Otorhinolaryngologists cannot look at this matter solely from the medical point-of-view. They must also consider the social construct of deafness - more specifically the public policies devised to improve the quality-of-life of the deaf - and actively participate in advocacy efforts for the deaf community.

The second dilemma deals with the consent required from parents/guardians to treat vulnerable patients with diminished autonomy who cannot decide for themselves. Informed consent forms must provide parents/guardians with thorough clarification on the medical aspects and risks related to cochlear implants, and describe the cultural implications the devices will produce upon their children's lives. This latter aspect is directly linked to the third dilemma: the potential extinction of the deaf culture in its wealth and intrinsic beauty.

The fourth dilemma verses on the criminal capacity of individuals equipped with cochlear implants. It is our belief that each case must be assessed individually with the aid of proper forensic examination to determine the degree of understanding the subject had of the criminal nature of his/her acts.

Lastly, the fifth dilemma: the role ENTs have to play in the matter. We believe that the decision on whether to implant cochlear hearing aids must be made considering the goal of offering deaf children an open future^2^, ^13^ while preserving their future rights of choice. Cochlear implants can ensure such future and, in these terms, may be regarded as a morally justified indication. ENT physicians have the moral duty and ethical obligation of offering their patients the best treatment available, providing parents/ guardians with information on all options available - and their pros and cons - without trying to influence them by acting in an unbiased manner and presenting opinions consistent with medical and scientific knowledge.

## References

[1] Lester EB, Dawson JD, Gantz BJ, Hansen MR (2011). Barriers to the early cochlear implantation of deaf children. Otol Neurotol..

[2] Nunes R (2001). Ethical dimension of paediatric cochlear implantation. Theor Med Bioeth..

[3] Hardonk S, Bosteels S, Desnerck G, Loots G, Van Hove G, Van Kerschaver E (2010). Pediatric cochlear implantation: a qualitative study of parental decision-making processes in Flanders, Belgium. Am Ann Deaf..

[4] Steel KP (1998). A new era in the genetics of deafness. N Engl J Med..

[5] Kermit P (2010). Choosing for the child with cochlear implants: a note of precaution. Med Health Care Philos..

[6] Westerberg BD, Pijl S, McDonald M (2008). Ethical considerations in resource allocation in a cochlear implant program. J Otolaryngol Head Neck Surg..

[7] Levy N (2002). Reconsidering cochlear implants: the lessons of Martha's Vineyard. Bioethics..

[8] Sacks O (1991). Seeing Voices: A Journey into the World of the Deaf..

[9] Beauchamp TL, Childress JF (2001). Principles of Biomedical Ethics.

[10] Daniels N (1985). Just Health Care.

[11] Rawls J (1971). A theory of Justice.

[12] Gillon R (1985). Deontological foundations for medical ethics?. Br Med J (Clin Res Ed)..

[13] Deleuze G (1974). Lógica do Sentido.

[14] Hladek GA (2002). Cochlear implants, the deaf culture, and ethics: a study of disability, informed surrogate consent, and ethnocide. Monash Bioeth Rev..

[15] Hyde M, Power D (2006). Some ethical dimensions of cochlear implantation for deaf children and their families. J Deaf Stud Deaf Educ..

[16] Hyde M, Power D (2000). Informed parental consent for cochlear implantation of deaf children: social and other considerations in the use of the ‘bionic ear'. Aust J Soc Issues..

[17] Hintermair M, Albertini JA (2005). Ethics, deafness, and the new medical technologies. J Deaf Stud Deaf Educ..

[18] Barros FAM (2001). Direito Penal.

